# Conversion of cellulose and hemicellulose of biomass simultaneously to acetoin by thermophilic simultaneous saccharification and fermentation

**DOI:** 10.1186/s13068-017-0924-8

**Published:** 2017-10-10

**Authors:** Xiaojing Jia, Xiaowei Peng, Ying Liu, Yejun Han

**Affiliations:** 1 National Key Laboratory of Biochemical Engineering, Institute of Process Engineering, Chinese Academy of Sciences, Beijing, 100190 China; 2 University of the Chinese Academy of Sciences, Beijing, 100049 China

**Keywords:** Acetoin, *Bacillus subtilis*, Corncob, Thermophilic bacteria, Simultaneous saccharification and fermentation

## Abstract

**Background:**

Acetoin (3-hydroxy-2-butanone), the precursor of biofuel 2,3-butanediol, is an important bio-based platform chemical with wide applications. Fermenting the low-cost and renewable plant biomass is undoubtedly a promising strategy for acetoin production. Isothermal simultaneous saccharification and fermentation (SSF) is regarded as an efficient method for bioconversion of lignocellulosic biomass, in which the temperature optima fitting for both lignocellulose-degrading enzymes and microbial strains.

**Results:**

A thermotolerant (up to 52 °C) acetoin producer *Bacillus subtilis* IPE5-4 which simultaneously consumed glucose and xylose was isolated and identified. By compound mutagenesis, the mutant IPE5-4-UD-4 with higher acetoin productivity was selected. When fermenting at 50 °C in a 5-L bioreactor using glucose as the feedstock by strain IPE5-4-UD-4, the acetoin concentration reached 28.83 ± 0.91 g L^−1^ with the acetoin yield and productivity of 0.34 g g^−1^ glucose and 0.60 g L^−1^ h^−1^, respectively. Furthermore, an optimized and thermophilic SSF process operating at 50 °C was conducted for acetoin production from alkali-pretreated corncob (APC). An acetoin concentration of 12.55 ± 0.28 g L^−1^ was achieved by strain IPE5-4-UD-4 in shake flask SSF, with the acetoin yield and productivity of 0.25 g g^−1^ APC and 0.17 g L^−1^ h^−1^. Meanwhile, the utilization of cellulose and hemicellulose in the SSF approach reached 96.34 and 93.29%, respectively. When further fermented at 50 °C in a 5-L bioreactor, the concentration of acetoin reached the maximum of 22.76 ± 1.16 g L^−1^, with the acetoin yield and productivity reaching, respectively, 0.46 g g^−1^ APC and 0.38 g L^−1^ h^−1^. This was by far the highest acetoin yield in SSF from lignocellulosic biomass.

**Conclusions:**

This thermophilic SSF process provided an efficient and economical route for acetoin production from lignocellulosic biomass at ideal temperature for both enzymatic hydrolysis and microbial fermentation.

**Electronic supplementary material:**

The online version of this article (doi:10.1186/s13068-017-0924-8) contains supplementary material, which is available to authorized users.

## Background

Acetoin (3-hydroxy-2-butanone), with a natural pleasant cream aroma flavor, abundantly exists in dairy foods and some fruits. It was widely used as food flavor enhancer in food industry [1] and applied as an important intermediate in drug and chemical synthesis and multifunctional materials [2, 3]. Due to these important applications, it was classified as one of the 30 bio-based platform chemicals which should be given the higher priority to the development and utilization by the U.S. Department of Energy in 2004 [4]. In fact, the demand of acetoin has been increasing in recent years, and more attentions have been focused on its production methods. Currently, commercially available acetoin is mainly obtained from fossil feedstocks through chemical conversion of 2,3-butanediol (2,3-BD), butanone, or diacetyl [1], whereas these chemical processes are not environmentally friendly and large-scale production of acetoin was limited by the low conversion rate, low safety of food, restrictions of raw materials, and so on. Therefore, some efforts have been made to develop biotechnological production methods including enzymatic conversion and microbial fermentation [2]. Enzymatic catalyst also uses 2,3-BD or butanone as raw materials, whereas it is difficult to achieve the industrialization of acetoin production as the limitation of enzymes supply and activity. On the other hand, microbial fermentative process not only displays advantages in environmental-friendly sources of raw materials, but also has benefits in lowering productive costs.

It is worthwhile to note that in these fermentation studies glucose was used as the main fermentable sugar, which may not be economically viable in industry [5–8]. Thus, the second-generation biofuel feedstocks, such as the lignocellulosic materials (e.g., straws, corncob), molasses, soybean meal hydrolysate, and seaweed hydrolysate, could save the costs for acetoin production [9–13]. To improve the conversion efficiency of biomass, separate hydrolysis and fermentation (SHF) and simultaneous saccharification and fermentation (SSF) have been applied for biofuel and biochemical production. In the SHF process, the raw materials are separately pretreated and degraded by biomass-hydrolyzing enzymes to obtain enzymatic hydrolysates. Then fermentable sugars presented dominantly as glucose (C6) and xylose (C5) in the degradation products were further converted to series of bio-chemicals by microorganisms [2, 14]. Multiple enzymes are required to hydrolyze lignocellulose efficiently into soluble sugars; however, the performances of the enzymes are inhibited due to the accumulation of end products [15]. In order to alleviate this problem, the enzymatic hydrolysis is performed simultaneously with fermentation; the process was referred as SSF. Thus, the hydrolyzed sugars by enzyme actions continuously were converted to bio-products by the fermenting microorganisms, thereby enhancing the efficiency of enzymatic saccharification [16]. However, the temperature inconsistency of saccharification and fermentation has hindered the efficient utilization of biomass in mesophilic SSF process; therefore, thermophilic SSF performing at suitable temperature for both process is a desire.

In the present study, a thermotolerant strain *Bacillus subtilis* IPE5-4 with super capability of producing acetoin at 50 °C from both glucose and xylose was isolated and characterized. In addition, a mutant strain *B. subtilis* IPE5-4-UD-4 with enhanced acetoin productivity was obtained by compound mutagenesis. The physiology and acetoin production profile of the *B. subtilis* strains at elevated temperature were further investigated. Moreover, the thermophilic SSF performances of the *B. subtilis* strains for acetoin production from alkali-pretreated corncob (APC) at 50 °C were systematically exploited.

## Results

### Isolation and identification of thermotolerant acetoin-producing strains

Thirty-eight isolates with the ability to produce acetoin were obtained from the soil samples by colorimetric reaction screens on agar plates at 50 °C. Twenty of them showed high capacity of acetoin fermentation from glucose. The isolate named IPE5-4 was screened to have the highest acetoin yield (5.69 g L^−1^) with 20 g L^−1^ glucose at 50 °C for 4 days. The physiological characteristics and biochemical tests of strain IPE5-4 were summarized in Additional file 1: Table S1. The strain IPE5-4 was a Gram-positive rod-shaped bacterium with spore forming. Its colony was originally larger and milky white with the surface being wrinkled and dry after culturing on lysogeny broth (LB) medium for 1 days. It was also able to reduce nitrate and produce catalase. In addition, strain IPE5-4 could utilize wide range of carbohydrates, including lignocellulose-related sugars and polysaccharides, suggesting its potential to convert lignocellulose to valuable bio-based chemicals. Besides, the pH range for growth is 4.7–9.0 and no growth occurred at NaCl concentrations above 10%. Moreover, the strain could grow up to 52 °C, implying that it might be an attractive candidate for high-temperature fermentation. Considering these features, strain IPE5-4 matched the physiological and biochemical characteristics of *B. subtilis* [17].

Alignment in GenBank database with other published 16S rRNA gene sequences showed that strain IPE5-4 shared 100% sequence identity with a number of *B. subtilis* strain, which was in accordance to the biochemical tests. A phylogenetic tree was constructed on the basis of the 16S rRNA gene sequence of strain IPE5-4 and closely organisms, resulting to cluster strain IPE5-4 with member of *B. subtilis* species (Additional file 2: Figure S1). Based on the consistency of morphology, physiological and biochemical properties and 16S rRNA gene sequence analysis, strain IPE5-4 was identified as *B. subtilis*. The partial 16S rRNA gene sequence of strain IPE5-4 had been deposited to GenBank with accession number KX259126 and under the name of *B. subtilis* IPE5-4.

### Improving acetoin productivity of the strain IPE5-4 by compound mutagenesis

The strain IPE5-4 was mutated to improve its capacity of acetoin production by compound mutagenesis of ultraviolet ray (UV) coupled with diethyl sulfate (DES). Mutants were firstly induced by treating parent IPE5-4 cells with series doses of UV light that resulted in different lethality rates by controlling the irradiation time. After irradiation process, 52 survivors were isolated. A total of 9 strains with improved acetoin production, representing 17.31% of the survivors, were obtained through flask fermentation. These acetoin production improved strains were mainly isolated in irradiation time of 60 and 80 s, when the UV light produced 70–80% lethality rate. Among these positive mutants, strain IPE5-4-UV-17 with the highest acetoin production of 13.26 ± 0.79 g L^−1^ was chosen for the next mutagenesis. Afterwards, mutants were induced by treating strain IPE5-4-UV-17 with series doses of DES that produced different lethality rate by controlling the intervals time. After DES treatment, a total of 57 survivors were isolated, 11 strains of which with higher acetoin production than IPE5-4-UV-17 were obtained through flask fermentation. The treating condition of 40–50 min and lethality rate of 80% with DES showed significant effect on IPE5-4-UV-17. Among these positive mutants, strain IPE5-4-UD-4 produced the highest concentration of acetoin (18.77 ± 0.24 g L^−1^), which was 50.62% higher than that of IPE5-4. This mutant was also been confirmed to be genetically stable for over 16 generations and thus chosen for further examination.

### Producing acetoin from glucose and xylose in shake flask fermentation

**Fig. 1:**
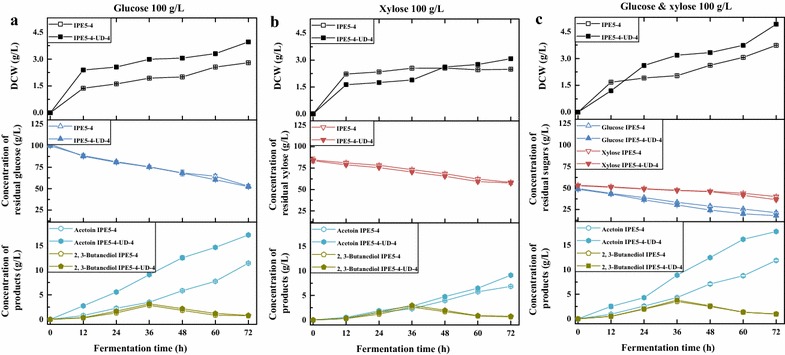
Time courses of acetoin production from glucose and xylose in shake flask fermentation. Both parent strain IPE5-4 and mutant strain IPE5-4-UD-4 were fermented with certain concentrations of sugars as carbon source.(**a**) Glucose 100 g L^−1^; (**b**) Xylose 100 g L^−1^; (**c**) Mixture of 50 g L^−1^ glucose and 50 g L^−1^ xylose). The cultivations were carried out in 100 mL (pH 7.0) medium at 50 °C and 200 rpm for 72 h

To further confirm the substrate utilization and metabolites production of the thermotolerant *B. subtilis* strains, both parent and mutant strains were fermented with highly concentrated glucose, xylose, or a mixture of both as carbon source at 50 °C for 72 h. Figure 1 demonstrates the time courses of acetoin production and glucose/xylose consumption in shake flask fermentation by the two strains. Taken together, the results suggested that both two strains displayed better preference and consumption for glucose than xylose in shake flask fermentation. It was worthwhile to note that significant change in carbon metabolism was observed for the mutant IPE5-4-UD-4. Firstly, the biomass of the mutant IPE5-4-UD-4 was evidently 41.60% (*P* < 0.001), 23.69% (*P* < 0.001), and 31.42% (*P* < 0.001) higher than those of the parent type using glucose, xylose, and mixed sugars, respectively. Secondly, the maximum consumption rate of glucose, xylose, and mixed sugar by the mutant were 22.97, 9.14, and 10.57% higher than those of the wild strain, respectively. Thirdly, significant enhancement of acetoin production by the mutant IPE5-4-UD-4 with glucose, xylose, and mixtures was observed, respectively, 50.15% (*P* < 0.001), 33.12% (*P* = 0.001), and 49.58% (*P* < 0.001) higher than those of the parent IPE5-4 after fermented for 72 h. In addition, in the mutant IPE5-4-UD-4 fermentation with the mixture of glucose and xylose (50 °C, 72 h), the concentration, yield, and productivity of acetoin reached 17.78 ± 0.19 g L^−1^, 0.38 g g^−1^, and 0.25 g L^−1^ h^−1^, respectively. The acetoin concentration, yield, and productivity were, respectively, 49.58% (*P* < 0.001), 31.03% (*P* = 0.002), and 47.06% (*P* < 0.001) higher than that of IPE5-4. Under the condition, by-products such as lactic acid, acetic acid, and 2,3-BD maintained at low levels as that of IPE5-4.

### Producing acetoin from glucose in a 5-L fermenter

**Fig. 2:**
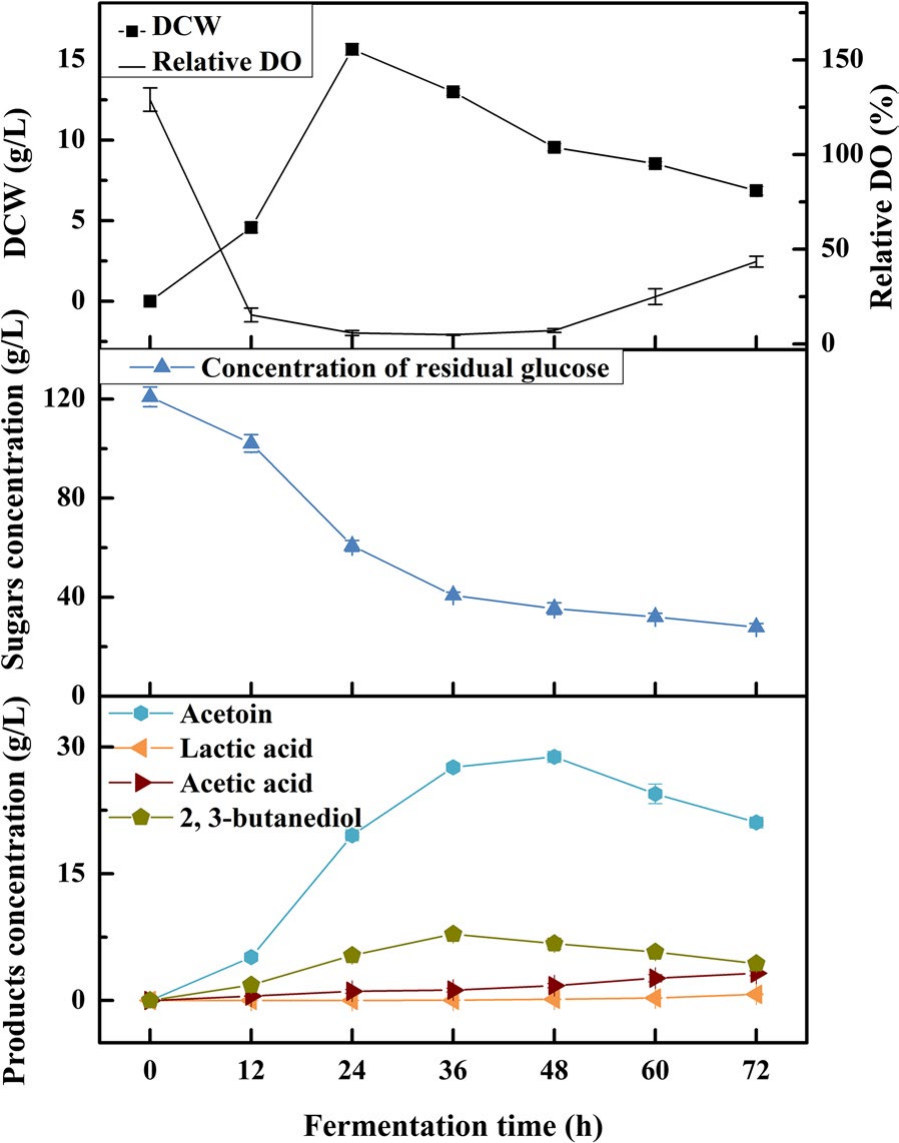
Time course of acetoin production from glucose by strain IPE5-4-UD-4 in a fermenter. The cultivation was conducted with 3 L medium containing ~ 120 g L^−1^ glucose at 50 °C for 72 h. The pH was maintained at 6.5 with stirring at 450 rpm with airflow of 0.6 vvm

The acetoin production ability of the mutant IPE5-4-UD-4 using glucose was further confirmed in a 5-L fermenter (Fig. 2). Maximum biomass was achieved after 24 h, with maximum glucose consumption rate of 2.51 g L^−1^ h^−1^. The dissolved oxygen (DO) nearly exhausted in 12 h, and maintained at a low level for a long time. Besides, acetoin was produced rapidly and the maximum concentration was detected at 48 h, while 2,3-BD occurred the highest yield at 36 h, as well as 72 h for lactic acid and acetic acid. Moreover, comparisons of the wild type and its mutant fermentation features with glucose are shown in Table 1. The consuming rate of glucose came to 1.78 g L^−1^ h^−1^ by mutant strain in a fermenter at shorted 48 h, which was respectively 61.24% (*P* = 0.001) and 63.48% (*P* < 0.001) higher than that for itself and the parent in shake flask. Similarly, the highest acetoin concentration (28.83 ± 0.65 g L^−1^) was obtained with a yield of 0.34 g g^−1^ glucose and productivity of 0.60 g L^−1^ h^−1^ when fermenting at 50 °C in a 5-L bioreactor, which was also significantly higher than that in the shake flask fermentation. Furthermore, the carbon ratios of C_Acetoin_/C_Glucose_ for the two strains were also analyzed, and the mutant IPE5-4-UD-4 was 42.42% higher than that of the parent in shake flask fermentation.

**Table 1:** Comparisons of acetoin production from glucose

Strain	IPE5-4	IPE5-4-UD-4	
Fermentation process	Shake flask	Shake flask	Fermenter
Substrate consumption			
Substrate concentration (g L^−1^)	99.92 ± 0.91	101.83 ± 3.81	120.94 ± 4.02
Substrate consumption time (h)	72	72	48
Concentration of residual substrate (g L^−1^)	52.98 ± 1.93	52.28 ± 2.19	35.38 ± 2.34
Consumption rate of substrate (g L^−1^ h^−1^)^a^	0.65	0.69	1.78
Acetoin production			
Maximum concentration (g L^−1^)	11.45 ± 0.11	17.20 ± 0.19	28.83 ± 0.65
Acetoin yield (g g^−1^)^b^	0.24	0.35	0.34
Acetoin productivity (g L^−1^ h^−1^)^c^	0.16	0.24	0.60
C_Acetoin_/C_Glucose_^d^	0.33 ± 0.01	0.47 ± 0.03	0.46 ± 0.02

^a^Consumption rate of substrate was calculated by [initial concentration of specific substrate (g L^−1^) − residual concentration of specific substrate (g L^−1^)]/substrate consumption time (h)

^b^Acetoin yield was calculated by g acetoin/g consumed substrate

^c^Acetoin productivity was calculated by maximum concentration of acetoin (g L^−1^)/substrate consumption time (h)

^d^ *C*_Acetoin_ meant the molar concentration of carbon in acetoin, and *C*_Glucose_ meant the molar concentration of carbon in glucose

### Producing acetoin from alkali-pretreated corncob in shake flask fermentation

**Fig. 3:**
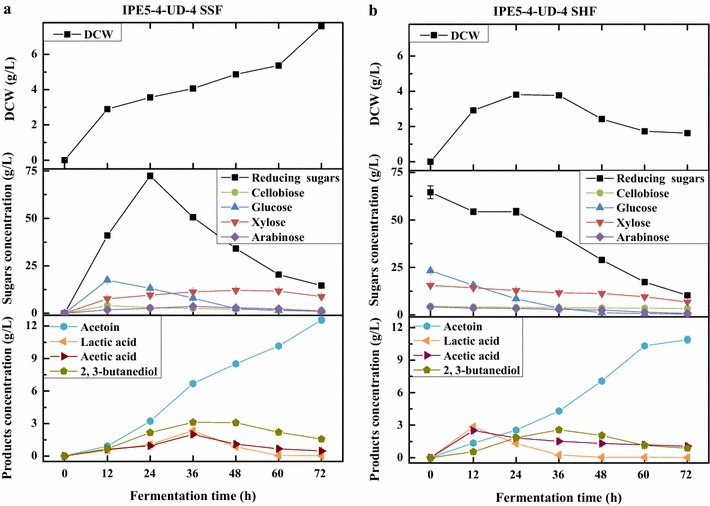
Time courses of acetoin production from APC in shake flask fermentation through SSF (**a**) and SHF process (**b**)

Based on the promising features of the mutant IPE5-4-UD-4, shake flask SSF and SHF processes were constructed to produce acetoin from APC. Figure 3 exhibited the time courses of acetoin production at 50 °C for 72 h. To sum up, the acetoin produced by IPE5-4-UD-4 via SSF was higher than that of SHF process. The maximum acetoin production, yield, and productivity were 12.55 ± 0.28 g L^−1^, 0.25 g g^−1^, and 0.17 g L^−1^ h^−1^, respectively, in SSF process at 50 °C for 72 h, which were 15.27% (*P* = 0.003), 15.14% (*P* = 0.003), and 15.23% (*P* = 0.006) higher than that of SHF. Meanwhile, the utilization of cellulose in the SSF approach reached to the maximum of 96.34%, as well as 93.29% for the use of hemicellulose, which were 2.04% (*P* = 0.020) and 52.59% (*P* < 0.001) higher than that for cellulose and hemicellulose through SHF process, respectively.

In the SSF approach (Fig. 3a), the cell growth straighten up during the whole fermentation. The total reducing sugars from APC accumulated at the beginning then gradually consumed accomplished with the similar trend of kinds of fermentable sugars. The accumulation of glucose and cellobiose peaked at 12 h, and decreased slowly in the rest period of the reaction, and almost cannot be detected after 48 h. The concentration of xylose and arabinose increased slowly at the first 36 h, and then decreased slightly during the reaction. Meanwhile, the acetoin production by the two strains increased continually and reached the maximum at the end of the fermentation. Simultaneously, the by-products concentrations of lactic acid, acetic acid, and 2,3-BD increased slowly at the first 36 h, and then declined to relatively low levels during the subsequent fermentation process, indicating the better acids utilization capacity of strain IPE5-4-UD-4.

In the SHF process (Fig. 3b), a total of 69.20 ± 0.78 g L^−1^ reducing sugars were generated from the APC after enzymatic hydrolysis and used for fermentation, containing 4.25 ± 0.01 g L^−1^ cellobiose, 29.66 ± 0.08 g L^−1^ glucose, 18.81 ± 0.14 g L^−1^ xylose, and 3.46 ± 0.12 g L^−1^ arabinose. The biomass reached maximum at 36 h then declined during the subsequent SHF process. As expected, the reducing sugars declined continually and corresponded to the rapid consumption of glucose. However, the amount of xylose and arabinose presented in the hydrolysate fell slowly during the whole SHF process. At the same time, the yield of acetoin increased linearly, and reached the maximum at the end of fermentation. The by-products lactic acid, acetic acid, and 2,3-BD also accumulated at earlier period, subsequently consumed and remained at lower level.

### Temperature optimization for SSF

**Fig. 4:**
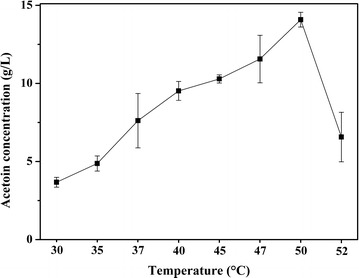
Temperature optimization for shake flask SSF. The process was performed using strain IPE5-4-UD-4 at 30–52 °C and 200 rpm for 72 h in 100 mL medium containing 5 g APC and 75 FPU cellulase

To evaluate the temperature profile for SSF process, which were conducted under different temperatures by using APC as substrate. As shown in Fig. 4, the acetoin yield at 50 °C was distinctly higher than that of the other temperature. Under the lower temperature (30–47 °C), the formation rate of acetoin increased with the rise of temperature. However, the acetoin titer at 52 °C declined significantly compared with that of 50 °C, which was therefore chosen as the optimum temperature for SSF.

### Producing acetoin from alkali-pretreated corncob in a 5-L fermenter

**Fig. 5:**
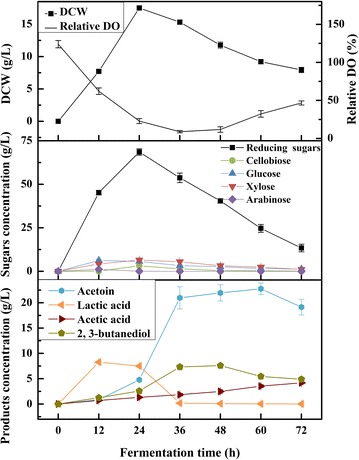
Time course of acetoin production from APC by strain IPE5-4-UD-4 in a fermenter. The cultivation was conducted with 3 L medium containing 5% (w/v) APC at 50 °C for 72 h. Cellulase at a final dosage of 15 FPU/g substrate was diluted, filter sterilized, and added along with seed-culture. The agitation speed was maintained at 200 rpm for the first 24 h, and then increased to 400 rpm with an airflow rate of 0.6 vvm. The pH was controlled at 6.5 by automatic adding 5 M HCl and 5 M NaOH

Moreover, the SSF ability of the mutant IPE5-4-UD-4 using APC to product acetoin was further confirmed in a 5-L bioreactor (Fig. 5). The cell growth and reducing sugars proceeded fast and reached the maximum at 24 h. The DO exhausted quickly in 24 h, and increased after fermentation for 48 h. Besides, the accumulation of fermentable sugars trended slowly at lower levels. In this case, the concentration of acetoin reached the maximum of 22.76 ± 1.16 g L^−1^ when fermenting at 50 °C in a 5-L bioreactor for 60 h, but finally decreased to 19.14 ± 1.56 g L^−1^ at 72 h. At the same time, 2,3-BD, as the primary by-product, increased to 7.59 ± 0.37 g L^−1^ at 48 h, but deduced to 4.90 ± 0.14 g L^−1^ at 72 h. However, the production of lactic acid raised to the highest of 8.27 ± 0.18 g L^−1^ at 12 h, then sharply fell to 0.18 ± 0.07 g L^−1^ at 36 h. Meanwhile, the concentration of acetic acid slowly rose to 4.19 ± 0.29 g L^−1^ after 72-h fermentation. In addition, comparisons of acetoin production from APC by strain IPE5-4-UD-4 are summarized in Table 2. The concentration of acetoin through SSF in a fermenter displayed significant elevation compared with that in shake flask fermentation. Moreover, the ratios of *C*_Acetoin_/*C*_APC_ in different fermentation processes were calculated, and fermenter SSF showed the highest acetoin carbon ratio per APC.

**Table 2:** Acetoin production from APC by strain IPE5-4-UD-4 through different processes

Fermentation process	Fermentation time (h)	Acetoin			
		Maximum concentration (g L^−1^)	Yield^a^ (g g^−1^)	Productivity^b^ (g L^−1^ h^−1^)	C_Acetoin_/C_APC_^c^
Shake flask SSF	72	12.55 ± 0.28	0.25	0.17	0.46 ± 0.01
Shake flask SHF	72	10.89 ± 0.34	0.22	0.15	0.42 ± 0.01
Fermenter SSF	60	22.76 ± 1.16	0.46	0.38	0.72 ± 0.04

^a^Acetoin yield was calculated by g acetoin/g substrate

^b^Acetoin productivity was calculated by maximum concentration of acetoin (g L^−1^)/fermentation time (h)

^c^C_Acetoin_ meant the molar concentration of carbon in acetoin, and C_APC_ meant the molar concentration of carbon in APC

## Discussion

Naturally, acetoin is excreted as an important intermediate metabolite by many microorganisms when they exist in environmental niche containing glucose or other fermentable carbon sources [14]. Currently, a variety of acetoin-producing bacterial strains had been isolated, such as the genus *Bacillus*, the lactic acid bacteria, the species *Paenibacillus polymyxa*, and the family Enterobacteriaceae [2]. Among these strains, *Bacillus* species are found to be excellent acetoin producers, and the fermentation process using glucose as carbon source has been reported with *B. subtilis* [5–8], *Bacillus amyloliquefaciens* [18, 19], and *Bacillus licheniformis* [20]. Remarkably, *B. subtilis* sub-strains could produce higher concentration of acetoin range from 11.45 to 43.80 g L^−1^ at 37 °C, which confirmed to have great potential in industrial application [5–8]. As a nonpathogenic bacterium, *B. subtilis* is one of the generally recognized as safe (GRAS) species, which has been widely used in industrial fermentation [2, 21]. Accordingly, extensive studies of the improvement of acetoin yield produced by *B. subtilis* strains had been paid more and more concerns.

In the present study, a positive mutant IPE5-4-UD-4 was screened by treating parent strain IPE5-4 with compound mutation of UV followed by DES. Significant enhancement had been made in the carbon metabolism of the mutant. Mutagenesis has been proven to be an effective method for improving the acetoin productivity of *B. subtilis* species. So far, *B. subtilis* TH-49 treated with random mutagenesis, produced the record acetoin (43.8 g L^−1^) in flask fermentation at 37 °C [8]. In addition, *B. subtilis* JNA-UD-6 engineered strain with 2,3-butanediol dehydrogenase (BDH) activity inactivation was treated by compound mutagenesis to produce 36.8 g L^−1^ acetoin in flask fermentation at 37 °C [6]. The acetoin-tolerant mutant *B. amyloliquefaciens* E-11, obtained by chemical mutagenesis and adaptive evolution, had a 25% increase of carbon flow to acetoin [19]. Although only a little evolution had occurred with the consumption rate of sugars by the mutant IPE5-4-UD-4, the carbon ratio and yield of acetoin changed dramatically when comparing with the wild strain in shake flask fermentation. Furthermore, the acetoin production was promoted to 28.83 g L^−1^ by strain IPE5-4-UD-4, with a yield of 0.34 g g^−1^ glucose and productivity of 0.60 g L^−1^ h^−1^ at elevated temperature for 48 h through culture in a fermenter at 50 °C, which was a remarkable record for acetoin production at high temperature.

The resulting effects of fossil fuels on environment and climate and economic dependency have obtained tremendous focus on utilizing the fermentable sugars from lignocellulosic biomass, the largest renewable carbohydrate source. Corncob is the main lignocellulosic by-product of sweet corn processing industry, thereby supplied in sufficient quantity without utilization in the harvested fields of China [22]. It has been considered as a prospective source for fermentable sugars which could be converted to bio-chemicals and fuels. However, as shown in Table 3, the acetoin yields (g g^−1^ dry biomass) from pretreated and un-pretreated lignocellulosic biomass through different methods were still at relatively low levels. Among them, the highest acetoin production to date (45.60 g L^−1^) was achieved by metabolically engineered *Enterobacter cloacae* in fed-batch fermentation of lignocellulosic hydrolysate of the pretreated corn stover at 37 °C [23]. Besides, high concentration of acetoin (19.00 g L^−1^) was also achieved by *Escherichia coli* mlc-XT7-LAFC-YSD utilizing saccharified cedar solution in batch SHF at 37 °C [24]. It was obvious that, in most cases, the lignocellulosic acetoin productions were performed with mesophilic microorganisms via SHF process at temperature around 37 °C due to the inconsistency of temperature for hydrolysis and fermentation.

**Table 3:** Acetoin production from lignocellulosic biomass by acetoin producers

Strain	Biomass	Fermentation process	Tem.^a^ (°C)	Con. (g L^−1^)	Pro.^b^ (g L^−1^ h^−1^)	Refs.
*B. subtilis* IPE5-4-UD-4	Pretreated corncob	Batch SSF, 5-L bioreactor	50; 50	22.76	0.38	This study
*K. pneumoniae* CICC 10011	Pretreated Jerusalem artichoke stalks	Batch SSF, 5-L bioreactor	37; 37	13.47	0.20	[9]
*E. coli*	Cellulose	Batch SSF, shake flask	37; 37	2.70	0.02	[13]
*E. cloacae* SDM 53	Pretreated corn stover	Fed-batch SHF, 7.5-L bioreactor	NA; 37	45.60	1.52	[23]
*K. pneumoniae* CICC 10011	Pretreated Jerusalem artichoke stalk and tuber	Fed-batch SHF, 5-L bioreactor	50–55; 37	11.40	0.16	[9]
*E. coli* DSM02-B	Pretreated brown seaweed	Fed-batch SHF, shake flask	50; 37	4.80	0.07	[12]
*E. coli* mlc-XT7-LAFC-YSD	Pretreated Japanese cedar wood chips	Batch SHF, shake flask	37; 30	19.00	0.16	[24]
*K. pneumoniae* CICC 10011	Pretreated Jerusalem artichoke stalk and tuber	Batch SHF, 5-L bioreactor	50–55; 37	11.80	0.23	[9]
Engineered *Zymomonas mobilis* 22C–BC5	Pretreated corn stover	Batch SHF, shake flask	50; 33	> 10.00	NA	[10]

*Tem.* temperature, *Con.* concentration, *Pro.* productivity, *Ref.* references, *NA* not available

^a^Temperature was indicated as the enzymatic saccharification temperature followed by the fermentation temperature

^b^Acetoin productivity was calculated by the concentration of acetoin (g L^−1^) presenting in the references/substrate consumption time (h)

Therefore, it is desirable to develop suitable fermentation strategies using cheap, nonfood, and renewable sources to obtain high acetoin yield and productivity. To this end, we investigated the efficiency of two principal process configurations which have been applied in the biotransformation of lignocellulosic biomass. Shake flask SSF performed better than SHF regarding to the increase in acetoin yield and productivity. It was likely that, in the enzymatic saccharification of SHF process, the biomass-hydrolyzing enzymes were subjected to the feedback inhibition of cellobiose and sugar monomers, which in turn decreased the efficiency of hydrolases and reduced the amount of fermentable sugars [16]. On the other hand, this limitation could be overcome by SSF process, hence making production of lignocellulosic acetoin to be economically feasible.

From an economic and technical standpoint, apart from low-price substrates, efficient strains are also required for industrial acetoin production. At present, industrial fermentation is typically conducted using mesophilic microorganisms such as lactic acid bacteria, fungi, and yeast, which grow optimally at a temperature range of 25–37 °C [25]. However, optimal saccharification of biomass to fermentable sugars by commercial cellulase is conducted at 50 °C. Hence, the temperature inconsistency of saccharification and fermentation has hindered the efficient utilization of biomass in SSF process. In this case, thermophilic SSF performing at the most suitable temperature for both saccharification enzyme and fermentation strain has advantages as it reduces the costs of the overall process (e.g., equipment costs, cooling costs, the costs of enzyme cocktail, and fermentation time costs), increases the efficiency of cellulase in the absence of product inhibition, and also decreases the contamination possibility [16]. To date, excellent acetoin productions by fermentation process are typically conducted at lower temperatures around 37 °C [5–8, 18–20]. Therefore, fermentation with an organism that matches the optimum condition for cellulase activity demonstrates great potential for enhancing products yield and efficiency. Herein, the physiology of *B. subtilis* strain IPE5-4 made it as the special case for efficient lignocellulosic acetoin production via SSF process at evaluated temperature. Surprisingly, up to 12.55 g L^−1^ acetoin was produced via shake flask fermentation with APC by the mutant strain IPE5-4-UD-4 at 50 °C, which was then promoted to the record of 22.76 g L^−1^ through SSF in a bioreactor.

It was worth noticing that fermentation with glucose and APC in a bioreactor performed especially better than in shake flask with respect to increase the acetoin yield and shorten the fermentation time. Acetoin formations by strain IPE5-4-UD-4 showed clearly positive correlations with the increase of oxygen supply with regard to constant aeration and agitation speed during the fermentation. Oxygen supply has been considered as an important limited factor for acetoin fermentation, affecting the product yield and by-product formation [2]. At the beginning, excessive oxygen supply generated much larger amounts of biomass. It has been reported that acetoin was excreted by *B. subtilis* with DO level above 100 parts per billion (ppb) [26]. Hereafter, under oxygen-limited conditions, the mixed acid occurred and acetoin converted into 2,3-BD due to the up-regulation of *alsSD* operon [27] and lower ratio of NAD^+^/NADH [28]. The consequently stage-enhanced agitation adopted in batch SSF process further ensured pretty good air supply and metabolic fluxes shift to acetoin fermentation in the late phase of fermentation [18].

## Conclusions

A thermotolerant acetoin producer *B. subtilis* IPE5-4-UD-4 was obtained by isolation and compound mutagenesis. The strain displayed superior acetoin productivity from both hexose and pentose at temperature up to 52 °C. It also displayed appealing performance in converting APC to acetoin through thermophilic SSF at 50 °C. The physiological and fermentation characteristics of the thermotolerant *B. subtilis* make it possible for efficient lignocellulosic acetoin production via thermophilic SSF at optimum temperature for both thermotolerant cellulase and fermentation strains. As the high titer of acetoin, understanding the genetic background of *B. subtilis* IPE5-4-UD-4 and application of which for strain breeding through genetic engineering will be further explored.

## Methods

### Reagent

Cellobiose, glucose, xylose, arabinose, acetoin, acetic acid, lactic acid, and other chemicals used were of analytical grade. Cellulase Cellic CTec2 (Novozymes, Tianjin, China) was used for enzymatic saccharification of corncob.

### Pretreating of raw materials

The corncob was harvested from local farm in Beijing, China. It was manually washed with tap water, collected, and dried at 60 °C. The dry corncob was pre-milled using a micro-grinder and sieved to 0.5–1.0 mm particle size fraction for the pretreatment. After that, the raw corncob was determined to contain 35.76% of cellulose, 25.32% of hemicellulose, and 22.09% of lignin. Then it was incubated with 2% (w/v) sodium hydroxide (NaOH) at a ratio of 1 g solid to 6 mL liquid and heated at 80 °C for 6 h at an agitation rate of 100 rpm. After treatment, the solids were collected by centrifugation and washed with tap water until the pH value reached neutral. Finally, the samples were dried at 60 °C to a constant weight and subjected for further analysis. The APC was determined to mainly compose of 35.76% of cellulose, 25.32% of hemicellulose, and 22.09% of lignin.

### Isolating and identifying thermotolerant strains for acetoin production

The soil samples from Dongying Oilfield (Shandong Province, China) were used for the isolation of thermotolerant acetoin-producing strains. Isolation medium containing peptone 10, yeast extract 5, NaCl 10, glucose 50 (g L^−1^, pH 7.0) was used in this study. For bacterial isolation, soil samples (5 g) were firstly re-suspended in 100 mL sterilized water by shaking at 200 rpm for 1 h. The suspension was then diluted with sterilized water, and 100 μL of the dilution was streaked onto agar medium and cultured at 50 °C for 1 day. The produced acetoin was roughly assessed by Voges-Proskauer reaction with slight modification [29]. The agar plates were firstly incubated with 0.5% creatine, and then washed with an equal volume of α-naphthol which dissolved in 2.5 M NaOH at 50 °C for 15 min. Single colonies which were larger in diameters and presented deeper red color were chosen and then confirmed in liquid culture at 50 °C for 4 days. The products were finally measured by colorimetric reaction at 535 nm with acetoin as a standard. The physiological and biochemical characterization of the selected strain IPE5-4 was carried out according to Bergey’s Manual of Systematic Bacteriology (2nd edition) [17]. The microbial morphology was examined by transmission electron microscope (Hitachi, Tokyo, Japan). Molecular identification of the strain was performed by 16S rRNA gene sequence analysis. An approximately 1.5-Kb sequence of bacterial 16S rRNA gene was successfully amplified by using the universal primer set 27F and 1492R. The PCR products were then purified and sequenced by Sangon (Shanghai, China). The partial 16S rRNA sequence of strain IPE5-4 was further analyzed with the online NCBI BLAST program (http://blast.ncbi.nlm.nih.gov/Blast.cgi?PROGRAM=blastn&PAGE_TYPE=BlastSearch&LINK_LOC=blasthome). The phylogram was created in MEGA 5.05 by the neighbor-joining method with 1000 bootstraps [30].

### Selecting mutant strains induced by compound mutagenesis

Strain IPE5-4 was mutated by UV coupled with DES based on the method reported by Zhang et al. [6]. For UV irradiation, cell suspension (about 10^7^ cells per mL) of the parent strain was exposed to an ultraviolet lamp (280 nm) at a distance of 25 cm and stirred with magnetic stirrer for different intervals (20 and 180 s). All of the treated and untreated cells were suitably diluted in sterile physiological saline. Hereafter, 200 μL of the cells was spread on to the isolation medium to calculate the lethality rate. Then samples with 70–80% lethality rate were subjected to the following isolation. Plates were incubated at 50 °C for 2 days and single mutants were further selected based on the yield of acetoin through shake flask fermentation.

For DES mutagenesis, cell suspension (about 10^7^ cells per mL) of the UV mutated strain was treated with 1% (w/v) DES in phosphate buffer (pH 7.0). After incubation at 37 °C for different intervals (between 10 and 60 min), the procedure was terminated by adding 25% (w/v) sodium thiosulfate. All of the treated and untreated cells were suitably diluted in sterile physiological saline. Hereafter, 200 μL of the cells was spread onto the isolation medium to calculate the lethality rate. Then samples with 80% lethality rate were subjected to the following isolation. Plates were incubated at 50 °C for 2 days and acetoin high-yielding single mutants were further selected based on the yield of acetoin through shake flask fermentation.

### Producing acetoin from glucose and xylose in shake flask fermentation

The capacity of the strains to utilize sugars was tested with different concentrations of glucose, xylose, and the mixture of both. For shake flask fermentation, the LB mediums supplemented with approximately desired concentrations of sugars (glucose or xylose ~ 100 g L^−1^; the mixture of 50 g L^−1^ glucose and 50 g L^−1^ xylose) were used for both wild and mutant. The fermentations were carried out at 200 rpm in 250-mL Erlenmeyer flasks with working volumes of 100 mL (pH 7.0). Aliquots of samples were withdrawn at 12-h intervals to determine the concentrations of biomass, residual sugars, and products.

### Producing acetoin from glucose in a 5-L fermenter

Culture was further performed with strain IPE5-4-UD-4 in a 5-L bioreactor (BIOTECH-5 JG, Baoxing Biological Equipment Engineering Co., Ltd, Shanghai, China). The operating volume of medium was 3 L and contained ~ 120 g L^−1^ glucose. The pH was controlled at 6.5 by automatically adding 5 M HCl and 5 M NaOH. And the agitation speed was set at 450 rpm with airflow at 0.6 volume of air/volume of medium/min (vvm). For seed-culture preparation, bacteria were cultivated in LB medium (pH 7.0) at 50 °C with shaking at 200 rpm for 12 h. Then 2% (v/v) of the seed-culture was transferred individually into fresh liquid fermentation medium and incubated at 50 °C for 72 h. Samples were taken periodically to determine the concentrations of biomass, residual sugars, and products.

### Producing acetoin from alkali-pretreated corncob in shake flask fermentation

To compare the acetoin production from APC by the strain IPE5-4-UD-4 via SSF and SHF, fermentations were performed in 250-mL Erlenmeyer flasks containing 100 mL of medium with shaking at 200 rpm on a rotary shaker. The thermophilic procedures were schematically shown in Additional file 3: Figure S2. For shake flask SSF, the fermentation medium contained the following (g L^−1^): APC 50, peptone 1, yeast extract 5, KH_2_PO_4_ 5, (NH_4_)_2_SO_4_ 2, and MgSO_4_·7H_2_O 0.2. Cellulase at a final dosage of 15 FPU/g substrate was diluted, filter sterilized and added along with seed-culture. For shake flask SHF, Erlenmeyer flask (250 mL) containing 100 mL citrate–phosphate buffer (50 mM, pH 7.0), 5 g APC, and cellulase at a final concentration of 15 FPU/g substrate, was incubated at 50 °C with 100 rpm for 72 h. After centrifugation, the supernatant of hydrolysis was filter sterilized and used as carbon source. The fermentation mediums were made up with (g L^−1^) peptone 1, yeast extract 5, KH_2_PO_4_ 5, (NH_4_)_2_SO_4_ 2, and MgSO_4_·7H_2_O 0.2. Samples were collected periodically to determine the concentrations of biomass, sugars, and products.

### Temperature optimization for SSF

To improve the acetoin production from APC by the strain IPE5-4-UD-4 via shake flask SSF, fermentations were performed at 30–52 °C and 200 rpm for 72 h. The 100 mL of medium containing the following components (g): APC 5, peptone 0.1, yeast extract 0.5, KH_2_PO_4_ 0.5, (NH_4_)_2_SO_4_ 0.2, MgSO_4_·7H_2_O 0.02, and 75 FPU cellulase was added in 250-mL Erlenmeyer flasks. The samples were taken at the end of reaction to determine the concentration of acetoin.

### Producing acetoin from alkali-pretreated corncob in a 5-L fermenter

Fermenter SSF with strain IPE5-4-UD-4 was conducted in a 5-L bioreactor with 3 L of initial medium which was the same with shake flask SSF. The agitation speed was kept at 200 rpm for the first 24 h, and then increased to 400 rpm with an airflow rate of 0.6 vvm. The initial pH was maintained at 6.5 by automatic addition of 5 M NaOH and 5 M HCl. The fermentations were carried out at 50 °C for 72 h and initiated individually by adding 2% (v/v) seed-culture which was prepared similarly as mentioned above. Aliquots of the liquid medium were collected periodically to determine the concentration of biomass, sugars, and products.

### Analytical methods

The chemical compositions of corncob after different treatments were determined according to the National Renewable Energy Laboratory analysis protocol (NREL/TP-510-42618). The total sugars presenting as the amount of reducing sugars were estimated by *para*-hydroxybenzoic acid hydrazide (PHBAH) method [31]. The dry cell weight (DCW) was calibrated against OD600. Cellobiose, glucose, xylose, arabinose, acetoin, 2,3-BD, acetic acid, and lactic acid generated from hydrolysis and fermentation process were measured by high-pressure liquid chromatography (HPLC) equipped with a Hi-PlexH exclusion column (300 × 7.7 mm, Agilent Technologies, United Kingdom) and a refractive index detector (LC-20AT, Shimadzu Corp., Japan). The injection volume was 10 μL, and 0.005 M H_2_SO_4_ was used as mobile phase. The column temperature was maintained at 65 °C and the flow rate was 0.6 mL/min.

### Statistical analysis

All of the experiments were performed in triplicates, and average and standard deviation (SD) values were adopted. Wherever necessary, data were analyzed by One-way ANOVA and Paired *T* test for test of statistical significances, and *P* < 0.05 was accepted.

## Additional files

**Additional file 1: Table S1.** Physiological characteristics of *B. subtilis* IPE5-4.

**Additional file 2: Figure S1.** Phylogenetic tree of strain *B. subtilis* IPE5-4 based on 16S rRNA gene sequences. The phylogenetic tree was constructed by neighbor-joining method using MEGA 5.05 with 1000 bootstraps. The bootstrap percentages were given at branch points. The GenBank accession numbers of 16S rRNA gene from different strains were listed after their names.

**Additional file 3: Figure S2.** Process diagram for acetoin production from APC in shake flask fermentation.

## Abbreviations

2,3-BD: 2,3-butanediol
SSF: simultaneous saccharification and fermentation
SHF: separate hydrolysis and fermentation
APC: alkali-pretreated corncob
GRAS: generally recognized as safe
BDH: 2,3-butanediol dehydrogenase
DO: dissolved oxygen
NaOH: sodium hydroxide
UV: ultraviolet ray
DES: diethyl sulfate
LB: lysogeny broth
vvm: volume of air/volume of medium/min
ppb: parts per billion
PHBAH: *para*-hydroxybenzoic acid hydrazide
DCW: dry cell weight
HPLC: high-pressure liquid chromatography
SD: standard deviation

## References

[1] Xiao Z, Lu JR (2014). Generation of acetoin and its derivatives in foods. J Agric Food Chem.

[2] Xiao Z, Lu JR (2014). Strategies for enhancing fermentative production of acetoin: a review. Biotechnol Adv.

[3] Zhu CJ, Tao S, Dong L, Wu JL, Yong C, Wang LF, Kai G, Ying HJ, Ouyang PK (2016). Production of liquid hydrocarbon fuels with acetoin and platform molecules derived from lignocellulose. Green Chem.

[4] Werpy T, Petersen G: Top value added chemicals from biomass: Volume I - Results of screening for potential candidates from sugars and synthesis gas. DOE/GO-102004-1992; TRN: US200427%%671; http://dx.doi.org/10.2172/15008859; 2004; p Medium: ED.

[5] Zhang X, Yang TW, Lin Q, Xu MJ, Xia HF, Xu ZH, Li HZ, Rao ZM (2011). Isolation and identification of an acetoin high production bacterium that can reverse transform 2,3-butanediol to acetoin at the decline phase of fermentation. World J Microbiol Biotechnol.

[6] Zhang X, Zhang R, Yang T, Zhang J, Xu M, Li H, Xu Z, Rao Z (2013). Mutation breeding of acetoin high producing *Bacillus subtilis* blocked in 2,3-butanediol dehydrogenase. World J Microbiol Biotechnol.

[7] Yixiao F, Yanjun T, Xiangying Z, Jiaxiang Z, Jianjun L (2013). Isolation of acetoin-producing *Bacillus* strains from Japanese traditional food-natto. Prep Biochem Biotechnol.

[8] Xu H, Jia S, Liu J (2011). Development of a mutant strain of *Bacillus subtilis* showing enhanced production of acetoin. Afr J Biotechnol.

[9] Li D, Dai JY, Xiu ZL (2010). A novel strategy for integrated utilization of Jerusalem artichoke stalk and tuber for production of 2,3-butanediol by *Klebsiella pneumoniae*. Bioresour Technol.

[10] Yang S, Mohagheghi A, Franden MA, Chou YC, Chen X, Dowe N, Himmel ME, Zhang M (2016). Metabolic engineering of *Zymomonas mobilis* for 2,3-butanediol production from lignocellulosic biomass sugars. Biotechnol Biofuels.

[11] Hespell RB (1996). Fermentation of xylan, corn fiber, or sugars to acetoin and butanediol by *Bacillus polymyxa* strains. Curr Microbiol.

[12] Mazumdar S, Lee J, Oh M-K (2013). Microbial production of 2,3 butanediol from seaweed hydrolysate using metabolically engineered *Escherichia coli*. Bioresour Technol.

[13] Shin H-D, Yoon S-H, Wu J, Rutter C, Kim S-W, Chen RR (2012). High-yield production of meso-2,3-butanediol from cellodextrin by engineered *E. coli* biocatalysts. Bioresour Technol.

[14] Xiao Z, Xu P (2007). Acetoin metabolism in bacteria. Crit Rev Microbiol.

[15] Jørgensen H, Kristensen JB, Felby C (2007). Enzymatic conversion of lignocellulose into fermentable sugars: challenges and opportunities. Biofuel Bioprod Bior..

[16] Choudhary J, Singh S, Nain L (2016). Thermotolerant fermenting yeasts for simultaneous saccharification fermentation of lignocellulosic biomass. Electron J Biotechnol.

[17] Rcw B, Sneath PHA, Mair NS, Sharpe ME, Holt JG (1986). Genus *Bacillus* Cohn 1872, 174^AL^. Bergey’s manual of systematic bacteriology.

[18] Zhang Y, Li S, Liu L, Jing W (2013). Acetoin production enhanced by manipulating carbon flux in a newly isolated *Bacillus amyloliquefaciens*. Bioresour Technol.

[19] Luo Q, Wu J, Wu M (2014). Enhanced acetoin production by *Bacillus amyloliquefaciens* through improved acetoin tolerance. Process Biochem.

[20] Liu Y, Zhang S, Yong Y-C, Ji Z, Ma X, Xu Z, Chen S (2011). Efficient production of acetoin by the newly isolated *Bacillus licheniformis* strain MEL09. Process Biochem.

[21] Maarten VDJ, Michael H (2013). *Bacillus subtilis*: from soil bacterium to supersecreting cell factory. Microb Cell Fact.

[22] Miura S, Arimura T, Itoda N, Dwiarti L, Feng JB, Bin CH, Okabe M (2004). Production of l-lactic acid from corncob. J Biosci Bioeng.

[23] Zhang L, Liu Q, Ge Y, Li L, Gao C, Xu P, Ma C (2016). Biotechnological production of acetoin, a bio-based platform chemical, from a lignocellulosic resource by metabolically engineered *Enterobacter cloacae*. Green Chem.

[24] Nakashima N, Akita H, Hoshino T (2014). Establishment of a novel gene expression method, BICES (biomass-inducible chromosome-based expression system), and its application to the production of 2,3-butanediol and acetoin. Metab Eng.

[25] Abdel-Banat BMA, Hoshida H, Ano A, Nonklang S, Akada R (2010). High-temperature fermentation: how can processes for ethanol production at high temperatures become superior to the traditional process using mesophilic yeast?. Appl Microbiol Biotechnol.

[26] Moes J, Griot M, Keller J, Heinzle E, Dunn IJ, Bourne JR (1985). A microbial culture with oxygen-sensitive product distribution as a potential tool for characterizing bioreactor oxygen transport. Biotechnol Bioeng.

[27] Frädrich C, March A, Fiege K, Hartmann A, Jahn D, Härtig E (2012). The transcription factor AlsR binds and regulates the promoter of the *alsSD* operon responsible for acetoin formation in *Bacillus subtilis*. J Bacteriol.

[28] Zhang L, Chen S, Xie H, Tian Y, Hu K (2012). Efficient acetoin production by optimization of medium components and oxygen supply control using a newly isolated *Paenibacillus polymyxa* CS107. J Chem Technol Biotechnol.

[29] Westerfeld W (1945). A colorimetric determination of blood acetoin. J Biol Chem.

[30] Tamura K, Peterson D, Peterson N, Stecher G, Nei M, Kumar S (2011). MEGA5: molecular evolutionary genetics analysis using maximum likelihood, evolutionary distance, and maximum parsimony methods. Mol Biol Evol.

[31] Lever M (1972). A new reaction for colorimetric determination of carbohydrates. Anal Biochem.

