# Supplementary material for: Conversion of cellulose and hemicellulose of biomass simultaneously to acetoin by thermophilic simultaneous saccharification and fermentation
Source: Biotechnol Biofuels. 2017 Oct 10;10:232. doi: 10.1186/s13068-017-0924-8 (PMC5635544; doi:10.1186/s13068-017-0924-8)
Supplement: Supplementary file 1 — Additional file 1: Table S1. Physiological characteristics of B. subtilis IPE5-4. [file 13068_2017_924_MOESM1_ESM.docx]

**Additional files 1: Table S1 Physiological characteristics of *B. subtilis* IPE5-4**

| **Characteristics** |  | **Strain IPE5-4** |
| --- | --- | --- |
| Morphology | Gram staining | + |
|  | Shape | Rod |
|  | Spore forming | + |
| Nutrient utilization | Nitrate reduction | + |
|  | Catalase | + |
|  | Voges-Proskauer test | + |
| Carbon utilization | Glucose | + |
|  | Xylose | + |
|  | Arabinose | + |
|  | Cellobiose | + |
|  | Lactose | + |
|  | Galactose | + |
|  | Mannose | + |
|  | Fructose | + |
| Polysaccharide degradation | Starch | + |
|  | Cellulose | + |
|  | Xylan | + |
| Physical growth conditions | pH | 4.7-9.0 |
|  | Temperature | Up to 52°C |
|  | Salt (NaCl) | 0-10% |
|  | Anaerobic | - |

Note: +, positive reaction; −, negative reaction
