# Supplementary material for: Conversion of cellulose and hemicellulose of biomass simultaneously to acetoin by thermophilic simultaneous saccharification and fermentation
Source: Biotechnol Biofuels. 2017 Oct 10;10:232. doi: 10.1186/s13068-017-0924-8 (PMC5635544; doi:10.1186/s13068-017-0924-8)
Supplement: Supplementary file 2 — Additional file 2: Figure S1. Phylogenetic tree of strain B. subtilis IPE5-4 based on 16S rRNA gene sequences. The phylogenetic tree was constructed by neighbor-joining method using MEGA 5.05 with 1000 bootstraps. The bootstrap percentages were given at branch points. The GenBank accession numbers of 16S rRNA gene from different strains were listed after their names. [file 13068_2017_924_MOESM2_ESM.docx]

**Additional files 2: Figure S1** Phylogenetic tree of strain *B. subtilis* IPE5-4 based on 16S rRNA gene sequences. The phylogenetic tree was constructed by neighbor-joining method using MEGA 5.05 with 1000 bootstraps. The bootstrap percentages were given at branch points. The GenBank accession numbers of 16S rRNA gene from different strains were listed after their names

Fig. S1
