# Supplementary material for: Conversion of cellulose and hemicellulose of biomass simultaneously to acetoin by thermophilic simultaneous saccharification and fermentation
Source: Biotechnol Biofuels. 2017 Oct 10;10:232. doi: 10.1186/s13068-017-0924-8 (PMC5635544; doi:10.1186/s13068-017-0924-8)
Supplement: Supplementary file 3 — Additional file 3: Figure S2. Process diagram for acetoin production from APC in shake flask fermentation. [file 13068_2017_924_MOESM3_ESM.docx]

**Additional files 3: Figure S2** Process diagram for acetoin production from APC
